# Pre-exposure cognitive performance variability is associated with severity of respiratory infection

**DOI:** 10.1038/s41598-022-26081-6

**Published:** 2022-12-30

**Authors:** Yaya Zhai, P. Murali Doraiswamy, Christopher W. Woods, Ronald B. Turner, Thomas W. Burke, Geoffrey S. Ginsburg, Alfred O. Hero

**Affiliations:** 1 Department of Computational Medicine and Bioinformatics, University of Michigan, Ann Arbor, MI 48109 USA; 2 Departments of Psychiatry and Medicine, Duke University School of Medicine, Durham, NC 27705 USA; 3 Duke Center for Applied Genomics and Precision Medicine, Duke University Medical Center, Durham, NC 27708 USA; 4 Department of Pediatrics, University of Virginia School of Medicine, Charlottesville, VA 22908 USA; 5 All of Us Research Program, National Institutes of Health, Bethesda, MD 20892 USA; 6 Department of Electrical Engineering and Computer Science, Department of Biomedical Engineering, and Department of Statistics, University of Michigan, Ann Arbor, MI 48109 USA

**Keywords:** Computational biology and bioinformatics, Biomarkers, Mathematics and computing

## Abstract

Using data from a longitudinal viral challenge study, we find that the post-exposure viral shedding and symptom severity are associated with a novel measure of pre-exposure cognitive performance variability (CPV), defined before viral exposure occurs. Each individual’s CPV score is computed from data collected from a repeated NeuroCognitive Performance Test (NCPT) over a 3 day pre-exposure period. Of the 18 NCPT measures reported by the tests, 6 contribute materially to the CPV score, prospectively differentiating the high from the low shedders. Among these 6 are the 4 clinical measures digSym-time, digSym-correct, trail-time, and reaction-time, commonly used for assessing cognitive executive functioning. CPV is found to be correlated with stress and also with several genes previously reported to be associated with cognitive development and dysfunction. A perturbation study over the number and timing of NCPT sessions indicates that as few as 5 sessions is sufficient to maintain high association between the CPV score and viral shedding, as long as the timing of these sessions is balanced over the three pre-exposure days. Our results suggest that variations in cognitive function are closely related to immunity and susceptibility to severe infection. Further studying these relationships may help us better understand the links between neurocognitive and neuroimmune systems which is timely in this COVID-19 pandemic era.

## Introduction

Cognitive function and other psychological factors (e.g. stress) have long been associated with physiological health. In particular, reaction time, vigilance and processing speed are central to the human ability to perform optimally. Accumulating evidence suggests that intra-individual variability in reaction time (and other cognitive domains) may reflect neurobiological disturbance and have valuable prognostic significance^1^. Higher variability of reaction time has been associated with greater mortality over 19-years of follow up in both younger and older adults as well as risk for falls and neurodegenerative disorders^2,3^.

Cognitive function is also closely linked to immune health and there is increasing recognition that immune cells play a physiological role in cognition and stress response^4^. For example, T-cells have been reported to have a pro-cognitive effect and neurotransmitters involved in the immune response, such as acetylcholine, dopamine and noradrenaline, also play a key role in cognition^4^. In healthy aging adults, elevated concentrations of pro-inflammatory cytokines has been linked to worse cognition^5^. This relationship is further reflected by the fact that many of the same factors that impair immune response (e.g. sleep deprivation, stress, alcohol consumption, depression, infections) also impair cognitive performance. Furthermore, several observational studies suggest that brain health, and its behavioral consequences, could be antecedent risk factors for infection. In particular, recently a large retrospective study of electronic health network data found that people with a history of psychiatric illness have a higher risk of being diagnosed with COVID-19^6^.

Using data from a challenge study, this paper shows that intra-individual variability in vigilance and reaction time measured over a 3 day baseline, reflecting subtle changes in immune and brain health, is associated with vulnerability to a common infection, the common cold (HRV). More specifically, in the context of a longitudinal human viral challenge study, we establish associations between pre-exposure cognitive function and post-exposure immune response, as measured by various markers, such as severity of symptoms and viral shedding. Among the pre-exposure cognitive markers studied here, we find that it is a new measure, the cognitive performance variability (CPV) score, that is most correlated to post-exposure immune response. The CPV score is extracted from a person’s performance on a web-based, computerized test battery called the NeuroCognitive Performance Test (NCPT) over 3 days leading up to exposure. The NCPT has previously been validated in a large sample of over 130000 normal volunteers^7^. For this study, we used 4 subtests of the NCPT designed to measure attention, processing speed, response inhibition and cognitive load (task switching and executive function)—domains known to be sensitive to fatigue, stress and infections to measure specific domains of a person’s cognitive performance.

**Figure 1:**
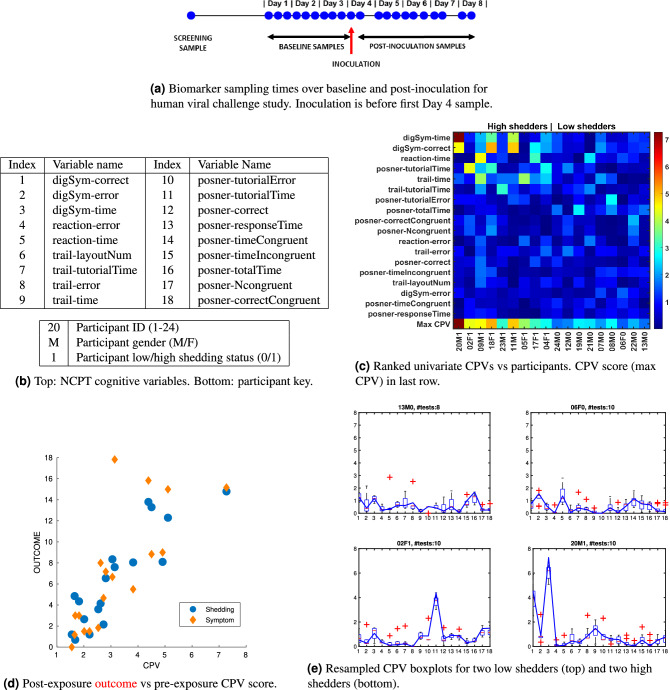
Pre-exposure cognitive performance variability (CPV) score is strongly associated with post-exposure outcome. (**a**) Challenge study layout showing baseline and post-inoculation biomarker sampling times for a study participant. (**b**) Definition of 18 NCPT variables measuring participant performance on 4 different tests: Digital Symbol Coding (DigSym), Go/No-Go (Reaction), Trail Making (Trail) and Attention Cuing (Posner). (**c**) Heatmap of univariate CPV scores for each study participant. participants are ordered from left to right in terms of decreasing amounts of post-exposure shedding. NCPT measures are ordered from top to bottom in terms of decreasing CPV max value over the participants. (**d**) Scatterplot of CPV and outcome for the 18 participants. Shedding (log$_{10}$ TCID$_{50}$/ml) and symptom (modified Jackson score) are aggregated over the full post-exposure period of the study. (**e**) Boxplots of the resampled univariate CPV scores, computed by successively leaving out a session, over the 18 NCPT variables in Fig. 1b for two of the lowest shedding (top row) and two of the highest shedding (bottom row) challenge study participants. Solid blue curve indicates the CPV scores shown in heatmap (**c**).

The set of 18 NCPT variables is shown in Fig. 1b. The CPV score is a measure of the person’s cognitive dissonance over time along any dimension. Unlike other measures of variability, like the linear coefficient of variation (CoV), the CPV score is a non-linear max-pooled measure of variability of the NCPT variables.

The main contribution of this paper is the demonstration that a certain kind of cognitive variability measure, the aforementioned CPV score computed from pre-exposure data, has an uncommonly strong association with a participant’s amount of post-exposure viral shedding and symptom severity. A sensitivity analysis shows that this score can be defined with as few as 6 of the 18 measures, 4 of which have recognized clinical significance.

## Results

A longitudinal viral challenge study was performed in 2015 in which 18 human volunteers participated over a period of 8 days (Fig. 1a). On the fourth day of the study participants were inoculated with human rhinovirus (HRV), the common cold, and the participants’ daily viral shedding and self-reported symptoms were collected for the remainder of the study. The cognitive function of the volunteers was collected three times per day over the pre-exposure days and the time series of 18 NCPT variables listed in Fig. 1b was transformed to a CPV score for each participant (see “Materials and methods”).

**Figure 2:**
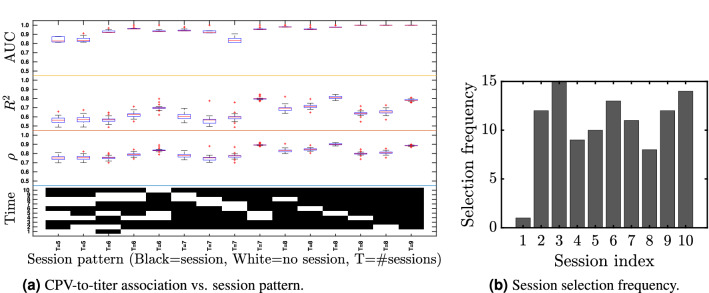
Lumos session timing patterns corresponding to the 15 highest CPV vs shedding correlations. Left: Boxplots of association measures between CPV and shedding for which the cross-validated (leave-one-participant-out) CPV Pearson correlation coefficients are at least 0.69 (the lower endpoint of 95% CI of correlation in Fig. 1d). The measures of association are the Pearson correlation coefficient $\rho$, the $R^2$ of linear regression, and the AUC of logistic regression of titers onto CPV. The AUC measures the association of CPV with Low vs High shedding (0,1) labels, where Low and High denote shedding below or above the population median, respectively. The pattern heatmap at bottom indicates the corresponding timing patterns of Lumos sessions with Time 1 corresponding to the initial screening and Time 10 corresponding to the test right before exposure. The number of sessions in each pattern is denoted by *T*, fewer than $T=7$ sessions significantly reduces the association. Right: relative frequency of inclusion of session times $1, \ldots 10$ in (**a**) (summed over session patterns).

Our main finding is that cognitive variability tracks severity of infection, as measured by both viral shedding and symptom severity, as shown in Fig. 1c–e. In these figures the cognitive variability was assessed by CPV using all baseline NCPT sessions excluding the initial (screening) session. The heatmap in Fig. 1c) shows that there are only a few NCPT variables exhibiting appreciable variation (univariate CPV) and that this variation is significantly higher for the higher shedding participants. This is especially evident in the CPV score, equal to the maximum of the univariate CPV’s, shown on the last row of the heatmap. Figure 1d shows a remarkably strong association of cognitive performance variability (CPV score) with both total amount of post-exposure shedding (Titers) and symptom severity (modified Jackson score). The Pearson correlation of CPV score and shedding(symptom) is 0.88(0.76) with pvalue (Fisher test) equal to $2\times 10^{-6}$($3\times 10^{-4}$).

While shedding and symptom may not be closely linked in general, we found total shedding and symptom severity to be highly correlated (Pearson 0.81, Supplementary Fig. S1). Furthermore, with one exception, low shedding implied low symptom severity and vice versa. Thus associations found between shedding and pre-inoculation biomarkers like the CPV are also present in symptom severity, although to a lesser degree. Therefore in the rest of this section we report associations for the less noisy shedding measurements. The total variance explained ($R^2$) by a linear model relating CPV score to shedding titers is $R^2=0.77$ (ratio of residual variance of linear regression to variance of titers). Furthermore, a logistic regression of total shedding onto the CPV score yielded a perfect discriminant between high and low shedders, respectively defined as those whose total shedding is below versus above the population median.

The correlation between shedding titers and CPV scores is robust to reductions in the number of NCPT variables composing the score. In fact the correlation between shedding and CPV increases to greater than 0.9 when only 6 NCPT measures are incorporated: digSym-time, digSym-correct, reaction-time, posner-tutorialTime, trail-time and trail-tutorialTime. Furthermore, the CVP score incorporating only the three basic NCPT measures digSym-time, digSym-correct, trail-time achieves a correlation level of approximately 0.7 (Fig. S2). We find that adding a fourth basic NCPT variable reaction time to the CPV score computation does not appreciably affect this level of correlation. On the other hand, replacing replacing either digSym-time or digSym-correct with posner-tutorialTime produces an increase in correlation to a level greater than 0.85.

To illustrate the role of the 18 individual NCPT variables in the CPV, we plot in Fig. 1e the univariate CPV scores for the two lowest shedding and the two highest shedding participants. This figure is extracted from Fig. S3 in the Supplementary that shows the sequence of univariate CPV scores for all 18 study participants. Superimposed on the plot of these variables is a boxplot indicating score sensitivity to session perturbation, determined by leave-one-out analysis where the univariate CPV was recomputed after successively leaving a single NCPT session out of each participant’s sequence (sans screening session). Figure 1e clearly shows that certain NCPT variables have significantly higher variability for the high shedders (lower two panels) than for the low shedders (top two panels). Note that the NCPT variable with highest variability (variable achieving peak score in each panel of Fig. 1e) differs across study participants.

As a point of comparison, our defined pre-exposure CPV score has considerably higher association to shedding than that attainable using the standard coefficient of variation (CV), whose correlation coefficient is less by factor of two (Pearson correlation $-\,\,0.42$ compared to 0.88) (Supplementary Fig. S4). Furthermore, while there is no discernable difference between low and high shedder distributions for the raw scores, such distributional differences are obvious for the CPV scores (See Supplementary Figs. S5 and S6). The CPV score has lower but statistically significant correlation with other clinically relevant cognitive variables over baseline (Supplementary Table S1). It has − 0.5 correlation with the standard deviation of sleep duration over baseline. It has 0.62 correlation with the Perceived Stress Score (PSS) assesed at screening time. It has 0.48 and − 0.46 correlation, respectively, with the mean and standard deviation of the Visual Analog Fatigue Score (VAFS) over baseline. However, the CPV was much more highly correlated with shedding titers than are other factors that have been previously related to susceptibility to infection, e.g., PSS (Supplementary Figs. S7 and S8).

To explore sensitivity to changes in the number of cognitive testing sessions and their timing, we performed a combinatorial study of the association between shedding and CPV as we vary both the number of NCPT sessions and their associated timing patterns over the baseline time period. As the number of sessions ranges from $T=3$ to $T=10$, Fig. 2 shows the top 15 patterns and their associations to infection severity as measured by correlation, $R^2$ and AUC.

**Table 1:** Pearson correlation $\rho$ between genes and 4 of the 5 top NCPT variables in Fig. 1c.

NCPT variable	#Gene correlations (FDR$<0.05$)	Top gene	$\rho$	P value	FDR
digSym_correct	33	LTF	0.515	5.93E−12	1.08E−07
digSym_time	23	ADGRG7	0.527	1.57E−12	2.86E−08
reaction_time	155	ADGRG7	0.527	1.58E−12	2.88E−08
posner_tutorialTime	9	MIR4760	0.654	2.14E−20	1.95E−16

Trail-time had no significant gene correlations (Fisher test at FDR 0.05) and is not shown. LTF (Lactotransferrin) is a Protein Coding gene that has been reported to stimulate the TLR4 signaling pathway leading to NF-kappa-B activation and subsequent pro-inflammatory cytokine production^8^. ADGRG7 (Adhesion G Protein-Coupled Receptor G7) is a Protein Coding gene that is found primarily in the intestine, but also in brain, cortex and cerebellum tissues^9^. MIR4760 is a miRNA with unknown function expressed in the brain and cortex^9^.

**Table 2:** Enriched pathways of genes that are significantly correlated with NCPT variables at FDR level 0.05.

Pathway Type	Name	FDR	Odds Ratio	Pathway size	Shared size
Transport and catabolism	Phagosome	3.99E−03	2.269	132	48
Digestive system	Protein digestion and absorption	4.90E−03	2.671	77	31
Digestive system	Cholesterol metabolism	4.90E−03	3.770	41	20
Transport and catabolism	Lysosome	6.46E−03	2.159	122	43
Infectious diseases: Viral	Influenza A	7.59E−03	1.965	154	51
Infectious diseases: Parasitic	Leishmaniasis	1.69E−02	2.605	63	25
Cancers: Overview	Proteoglycans in cancer	2.18E−02	1.810	166	52
Immune system	Chemokine signaling pathway	2.23E−02	1.794	167	52
Infectious diseases: Bacterial	Salmonella infection	4.78E−02	2.189	73	26
Infectious diseases: Viral	Kaposi sarcoma-associated herpesvirus infection	4.78E−02	1.695	177	53
Cancers: Overview	Pathways in cancer	4.78E−02	1.387	464	120
Development	Osteoclast differentiation	4.78E−02	1.839	123	39

More than half of these pathways are related to immune response.

We explored possible connections between the 5 most discriminating NCPT measures in Fig. 1c and gene expression. The sample correlation was computed between the baseline sequence of NCPT scores and the baseline sequence of RNAseq gene expression levels, obtained from peripheral blood assays. There are over 100 genes that are significantly correlated to four of the NCPT variables (Pearson’s correlation test at FDR $<0.05$) and some of these genes have associated FDR p-value less than $10^{-7}$ (Table 1). Among the top 5 NCPT variables, only Trail-time had no significant gene correlations. The correlation between digSym-correct and the gene LTF (Lactotransferrin) was highly significant (FDR $10^{-7}$). LTF is a Protein Coding gene that has been reported to stimulate the TLR4 signaling pathway leading to NF-kappa-B activation and subsequent pro-inflammatory cytokine production^8^. digSym-time and reaction-time had highly significant correlation (FDR $<3\times 10^{-8}$) with ADGRG7 (Adhesion G Protein-Coupled Receptor G7), which is a protein coding gene found primarily in the intestine, but also in brain, cortex and cerebellum tissues^9^. posner-tutorialTime was very significantly correlated (FDR $<10^{-15}$) with MIR4760, a miRNA with unknown function but primarily expressed in the brain and cortex^9^. A pathway enrichment analysis revealed that more than half of the discovered pathways (FDR $<0.05$) are relevant to immunity, including transport and catabolism, infectious disease, and immune system pathways (Table 2).

## Discussion

The 6 out of 18 major NCPT measures that contribute significantly to the CPV score collectively represent all 4 types of tests, suggesting that cognitive variability is related to immunity through a complex and novel combination of factors. Four of these 6 NCPT measures are time-to-completion measures and only one of them is a correctness measure. With the exception of posner-tutorialTime, these measures are common clinical measures used to test cognitive function of patients. In particular, digSym-correct and digSym-time are used to assess cognitive processing speed, working memory, visuo-spatial processing, and attention. The reaction-time measure is used to assess response inhibition and processing speed. The primary outcome of the Trail Making B test, trail-time, is used to assess visual ability, motor functioning, cognitive processes, and executive functioning. Of the 13 other NCPT measures, the control variable Trail-layoutNum, randomly generated at the start of each Trail test, has no influence on the final CPV score. Furthermore, we found that the high association between baseline cognitive performance variability and shedding disappears after viral inoculation (Supplementary Fig. S9) for which the distributions of low and high shedders’ CPV scores cease to be discriminating. These findings suggest that the infection distinctly perturbs both low and high shedders away from their quiescent pre-exposure cognitive states.

The combinatorial study shown in Fig. 2 indicates interesting structure in the session patterns that yield high correlation between the CPV and viral shedding. As might be expected, the association tends to decrease when the number of sessions decreases. Exclusion of the early initial screening session at time 1 tends to increase the correlation. Inclusion of the final baseline session at time 10 (right before inoculation) also tends to increase correlation. Interestingly, for $T=7$ if the last baseline session (time 10) is included then we can maintain the correlation above 0.7 only if we omit the screening session (time 1) and if the 2 other omitted sessions are successive. Indeed, all of the top 15 patterns have gaps between test times that do not exceed 16 hours. We also observe that a correlation greater than 0.7 (AUC $>0.8$) is attainable even when there are as few as $T=5$ sessions, as long as they are distributed such that there is at least one test on each of the three pre-exposure days.

One of the most prominent NCPT correlated genes, ADGRG7, encodes G protein-coupled receptor 128 (GPR128), a member of adhesion G protein-coupled receptor. Although there is no direct evidence connecting GPR128 and cognition, another member from the same family, GPR110, has been characterized as a potential target for controlling pathophysiological processes of neurodevelopment and function^10^. Many of the enriched pathways found in our analysis contain MAPKs. Dysregulation of the RAS/MAPK signaling cascade has been reported to be associated with severity of cognitive impairments in patients^11^. While our findings are based only on observational data, they suggest an interesting temporal connection between NCPT variables and the molecular mechanisms of cognition and immunity.

These findings raise the intriguing possibility that periodic cognitive testing for assessing susceptibility to severe infection may have clinical and/or epidemiological value. However, there are several factors that might impede translation of our results to the clinic or to public health. First, continuous cognitive testing over time would be necessary as the time of viral exposure cannot be anticipated. This could possibly be overcome by using a passive sensor-based continuous measurement of a person’s reaction time. Second, perhaps an algorithm that combines more easily collected stress, sleep and cognition measures could achieve equal or higher accuracy. It is unknown whether our results would replicate for a different pathogen or for people in a different demographic category than the young healthy participants in our study. This includes, in particular, older people in nursing facilities who are at higher risk but whose cognitive dysfunction may confound our ability to detect a signal.

We note that the viral challenge resulted in an unbalanced gender distribution over high and low ranges of shedding and symptom severity. In particular, almost all of the low shedders were men while close to half of the participants in the high shedding group were women, as were three of the four participants reporting the severest symptoms (Supplementary Fig. S1). The mean symptom severity(shedding) was 10.2(8.3) for women and 5.3(5.7) for men. While this imbalance could be due to chance it is also possible that this reflects gender-dependent immune response differences, as have been reported in other challenge studies^12^. Notably, it has been previously reported that females tend to have a higher number of symptoms than males when challenged with the flu^13^.

As in any observational study, there are limitations to our findings, including the small sample size (18) of our study. There was variation in the degree of compliance with the prescribed cognitive testing protocol. Four of the 18 participants completed fewer than 10 NCPT sessions and 2 participants did not participate in the NCPT early screening session. Furthermore, some participants did not abide strictly to the prescribed NCPT session timing (early morning, mid-day and evening). The concurrent infection of 3 of the participants with both wildtype and RV39 challenge viruses may be a confounding factor that could have been eliminated had all participants been isolated during the study. While our observational findings cannot establish that CPV or any of its correlates are causal factors for increased viral shedding, the reported associations suggest that cognitive performance variability deserves further study in the context of disease susceptibility and severity.

Our findings add to a growing literature pointing to possible advantages of cognitive performance variability measures as compared to raw cognitive scores. Recent studies have noted that there is a substantive short-term within-person variability in cognitive functioning, suggesting that single raw scores may be less informative about an individual’s true level of functioning^14^. This suggests the same raw units of measurement may not have the same meaning for everyone and that expressing change relative to one’s own across-occasion variability may have greater sensitivity for capturing subtle neurobiological disturbance. Indeed, a 19-year long longitudinal study^2,3^ reported that higher variability of reaction time was associated with greater mortality as well as risk for falls and neurodegenerative disorders. This raises the intriguing prospect of larger scale testing of CPV measures in longterm observational studies that may reveal significant associations between cognitive variability, immunity, and health.

**Figure 3:**
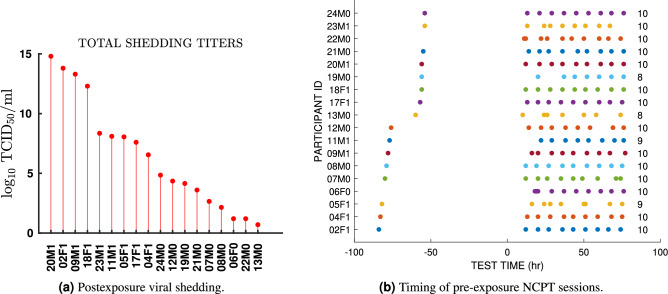
Total post-exposure viral shedding and pre-exposure NCPT session timing. (**a**) Total amount of viral shedding accumulated from the time of exposure to the end of the study, indexed over participant ID’s. The participant ID encodes the participant index (numeric first two characters from 1 to 20) the participant gender (M or F second character) and whether the participants’ shedding is below or above (0 or 1) the population median of total viral shedding (5.7 titers). (**b**) Timing of the pre-exposure NCPT testing sessions for each of the 18 challenge study participants during the baseline part of the challenge study (0–80 h), which precedes exposure to the HRV pathogen. Appearing on the far left of the figure are the participants’ initial screening sessions which, for all but two participants, occurred several days before the start of the study. At far right of the figure is shown the total number of sessions, varying between 8 and 10, for each participant.

## Materials and methods

### Challenge study protocol

The challenge study experiment and data collection described in this section were performed in accordance with relevant guidelines and regulations approved by the Internal Review Boards at Duke University^15^ and the University of Virgina^16^. Written informed consent was obtained from all study participants. A blank copy of the consent form is included in the repository^17^.

Among other biomarkers, viral shedding, symptoms, and cognitive performance data were collected from a human rhinovirus (HRV) challenge study (see Fig. 1a). The challenge study was designed by Duke University and University of Virginia. The study was performed at the University of Virginia in Charlottesville in mid-September 2015 and all participants were recruited from the University community. A total of 24 volunteers were recruited and 19 participated in the study. One of these participants had a failed inoculation and was omitted from our analysis. The age range of the remaining 18 participants was between 18 and 23, two thirds of these participants were male, and 4 were non-caucasian. For a more detailed demographic summary see Supplementary Fig. S10. The study protocol was reviewed and approved by the Institutional Review Boards at the University of Virginia and Duke University^15,16^. Written informed consent was obtained from all participants. Exclusion criteria included pregnancy, chronic respiratory illness, high blood pressure, tobacco/drug/alcohol history, and high serum antibody levels to RV39. All participants were screened prior to the study to ensure that they met the exclusion criteria. The participants were not isolated during the study.

The challenge study lasted 8 days over which various types of biomarkers were continuously collected from participants using wearable wristbands (Empatica E4), whole blood assays (RNAseq, steroids), nasal-pharyngeal washes (viral shedding), cognitive testing (Lumos), and self-reported clinical data (symptoms). Biomarkers were collected at a clinical site three times daily at roughly 8 hour intervals in the early morning, mid afternoon and late evening. Symptom scores were collected prior to blood draws and nasal procedures. Figure 1a represents the actual biomarker collection times for one of the participants.

On day four at approximately 8am (Fig. S11) each participant was inoculated via intranasal drops of diluted Human Rhinovirus strain type 39 with a dose of 100TCID50 in 1mL Lactated Ringer’s Solution. The IND number for the RV-39 challenge pool was 12934. Prior to inoculation a multiplex PCR was performed on all volunteers to detect unexpected respiratory pathogens, specifically, influenza, parainfluenza, picornavirus/RV, metapneumovirus, respiratory syncytial virus, adenovirus and coronavirus. There were four participants (#3,#4,#6,#13) who had wild virus detected and all were rhinovirus. One participant (#3) did not develop an infection with the RV39 challenge virus and was excluded from the data analysis. Three participants who had virus detected prior to inoculation went on to develop an infection with RV39 and were included in the data analysis. Starting on the day after inoculation the participants underwent daily nasal lavage each morning and the amount of viral shedding was determined by serial dilution in cell culture as described in the virus isolation section of^18^. The identity of the rhinovirus shed in nasal secretions was confirmed as RV39 in all cases by a typing neutralization assay using specific RV39 antiserum. Excluding participant #3, all volunteers entering the challenge study shed detectable RV39 titers on at least one day during the post-inoculation time period (Days 5 through 8). The total amount shedding of a participant is defined as the sum of all shedding titers collected over this time period. Figure 3a shows the total amount of shedding for each participant over all post-inoculation study days, ordered from maximum to minimum.

Participants recorded their symptoms in a symptom diary at each post-inoculation biomarker sampling time (Fig. 1a). To quantify symptom severity, we used the standardized modified Jackson score used in previous studies on respiratory infection^19,20^. Specifically, participants ranked 8 symptoms of upper respiratory infection (chills, cough, headache, nasalobstruction, runnynose, sneezing, sorethroat, tiredness) on a scale of 0–3, respectively corresponding to “no symptoms”, “just noticeable”, “bothersome but can still do activities” and “bothersome and cannot do daily activities.” The scalar modified Jackson score was then computed by summing all 8 rankings. These modified Jackson scores were converted into an average daily symptom score by averaging the scores recorded as daily diary entries. The total symptom score is the sum of the average daily scores over the 5 day post-inoculation time period.

Cognitive scores were collected in test sessions performed 3 times per day (early-morning, at mid-day, and late-evening) as shown in Fig. 3b for the pre-inoculation phase of study (see Supplementary Fig. S12 for full study). This data was also collected from a reference session prior to the start of the study. In each session the participant answered web-based questionnaires and engaged with Lumos brain testing software using computer tablets that were provided to them.

*NeuroCognitive performance test* The NeuroCognitive Performance Test (NCPT) is a repeatable, web-based, computerized, cognitive assessment platform designed to measure subtle changes in performance across multiple cognitive domains^7^. It comprises of 18 subtests and the modular platform allows for customized subtest batteries for specific studies. It was formerly referred to as the Brain Performance Test. As such computerized tests may offer several advantages over traditional paper and pencil methods, such as greater consistency in administration and scoring, generation of alternate forms for repeated testing, precise stimulus control, ability to capture and analyze multiple components of a test taker’s response, adaptation of difficulty levels, greater convenience and ability to administer at different settings. Test reliability and concurrent validity of the NCPT for unsupervised administration has been previously published. Specifically, the authors of^7^ reported normative data for more than 130,000 individuals aged 13–89 years as well as data on the ability of NCPT to detect mild cognitive impairments.

The specific NCPT battery used in the study comprised of four subtests designed to measure attention, processing speed, response inhibition and cognitive load (task switching and executive function)—domains known to be sensitive to fatigue, stress and infections^21^. The brief battery (15 minutes) was designed to be easy to complete and included the four subtests described below:

1. Attentional Cueing (Posner): A measure of selective attention and processing speed. An arrow cue is shown followed by a stimulus placed in one of 2 locations. participants pick the correct location of the stimulus.
2. Digital Symbol Coding: A measure of attention/vigilance, speed and immediate memory. participants enter the number corresponding to randomly generated symbols using a key at the top of the screen in 90 seconds. The primary measure is number of correct responses minus number of incorrect responses.
3. Go/No-Go: A measure of response inhibition and processing speed. Participants were required to respond as quickly as possible to a target, but to avoid responding to distractions.
4. Trail Making B: A measure of executive function, speed and mental flexibility. participants connect the numbers from smallest to largest alternating between numbers and letters. The primary measure is completion time and there is no time limit.

These 4 subtests yield 18 scores related to speed, accuracy and congruency. The tests were administered at 10 time points across 3 days at baseline. Raw scores on all 18 tests across all 10 time points were used to compute the cognitive variability indices, defined in Eq. (1) below, for each participant and NCPT variable.

In addition to NCPT, several well established self-reported psychometric markers were measured at various times in the study. This included responses to fatigue related questions using two protocols: the Visual Analog Fatigue Scale (VAFS), measured 3 times per day; and the Fatigue Severity Scale (FFS), measured at screening and on the fourth day of the study. The VAFS is a response to a single question scoring fatigue from 10 (no fatigue) to 0 (severe fatigue), while FFS is comprised of responses to 9 fatigue-related questions. A large scale clinical validation study of these measures of fatigue was reported in^22^. The Perceived Stress Scale (PSS) was used to measure stress at the initial screening session. The PSS is an instrument that measures a person’s perceived stress over the past month consisting of 10 questions about stress on a scale of 0–4, which has been clinically validated in^23^. Finally, the reduced Composite Scale of Morningness (rCSM) was used to measure an individual’s chronotype. The rCSM consists of a subset of 7 questions from the set questions of the full CSM^24^ on the most productive part of the day. The rCSM has been clinically validated in^25^.

### Cognitive performance variability score (CPV)

We quantify variation of within-participant cognitive function over baseline by using a max-pooled change statistic derived from the time sequences of baseline NCPT scores. For a particular participant *i* and cognitive variable *j*, e.g., NCPT *reaction-time*, let the value of the variable at the *m*-th session time be $x_{ij}(m)$, $m=1, \ldots , N_{ij}$, where $N_{ij}$ are the number of time samples, e.g, number of NCPT sessions, prior to inoculation time. Define the cognitive variability index $T_{ij} \in [0,1]$:

1 $$\begin{aligned} T_{ij}= & {} \frac{(N_{ij}-2)\sum _{m=2}^{N_{ij}} (x_{ij}(m)-x_{ij}(m-1))^2}{ (N_{ij}-2)\sum _{m=2}^{N_{ij}} (x_{ij}(m)-x_{ij}(m-1))^2 +(N_{ij}-1) \sum _{m=2}^{N_{ij}} (x_{ij}(m)-x_{ij}(m-1)-\overline{\Delta x}_{ij})^2}, \end{aligned}$$

where $\overline{\Delta x}_{ij}=(N_{ij}-1)^{-1}\sum _{m=2}^{N_{ij}}(x_{ij}(m)-x_{ij}(m-1))$ is the sample mean of the successive differences $\{x_{ij}(m)-x_{ij}(m-1)\}_{m=2}^{N_{ij}}$. For each participant *i*, $T_{ij}$ is a normalized measure of performance variability over successive sessions $m-1$ and *m* for the *j*-th NCPT variable. More specifically, we can interpret $T_{ij}$ as an analysis of variance (ANOVA) test statistic for testing the null hypothesis that there is no change in mean cognitive performance over successive sessions. Under this null hypothesis the differences $x_{ij}(m)-x_{ij}(m-1)$, $m=2, \ldots , N_{ij}$, have zero mean and a method-of-moments estimate of the variance of these differences is their sample second moment $\sigma _0^2=(N_{ij}-1)^{-1}\sum _{m=2}^{N_{ij}} (x_{ij}(m)-x_{ij}(m-1))^2$. On the other hand, under the alternative hypothesis the method-of-moments estimate of the variance is $\sigma _1^2=(N_{ij}-2)^{-1} \sum _{m=2}^{N_{ij}} (x_{ij}(m)-x_{ij}(m-1)-\overline{\Delta x}_{ij})^2$, which is the sample second moment of the differences centered about their sample mean. The cognitive variability index can thus be written $T_{ij}=\sigma ^2_0/(\sigma ^2_0+\sigma ^2_1)$ and, as $\sigma ^2_0/\sigma ^2_1$ increases in the magnitude of the mean variability $|\overline{\Delta x}_{ij}|$, $T_{ij}$ is a natural measure of performance variability. Assuming the successive differences are independent Gaussian, under the null hypothesis $T_{ij}$ has a Beta distribution with parameters $N_{ij}-1$ and $N_{ij}-2$, giving the following expression for the – log p-value:

$$\begin{aligned} CPV_{ij}= -\log \left( 1-\frac{B\left( T_{ij},\frac{N_{ij}-1}{2}, \frac{N_{ij}-2}{2}\right) }{B\left( \infty ,\frac{N_{ij}-1}{2}, \frac{N_{ij}-2}{2}\right) } \right) \end{aligned}$$

where *B*(*x*; *a*, *b*) is the incomplete beta function

$$\begin{aligned} B(x;a,b)=\int _0^x t^{a-1}(1-t)^{b-1} dt. \end{aligned}$$

The CPV score for the *i*-th participant is defined as the maximum

$$\begin{aligned} CPV_i=\max _{j}CPV_{ij}. \end{aligned}$$

$CPV_{ij}$ can be computed by applying the one-sided ANOVA significance testing procedure^26^ to the columns of the matrix

$$\begin{aligned} {\mathbf {X}}_{ij}= \left[ \begin{array}{ccc} x_{ij}(1) &{}\ldots &{} x_{ij}(N_{ij}-1) \\ x_{ij}(2) &{}\ldots &{} x_{ij}(N_{ij}) \end{array} \right] . \end{aligned}$$

This mathematical equivalence allows us to compute the CPV using standard ANOVA software (Matlab R2020a anova1.m).

*RNA assays* Blood draws were collected from the participants three times per day in the morning, afternoon and late evening according to a time sampling protocol represented in Fig 1d. Following standard extraction procedures from whole blood, we used whole transcriptome shotgun sequencing (RNAseq) to characterize peripheral blood mRNA. Total RNA was extracted from PAXgene blood samples, and quality was assessed using the Nanodrop UV spectrophotometer and Agilent 2100 Bioanalyzer. Then abundant ribosomal RNA and globin transcripts were depleted, RNA was converted to cDNA for library preparation (cDNA fragmentation, adapter ligation and PCR, QC check), which was hybridized to flow cell for sequencing by synthesis on the Illumina HiSeq2000 platform. The short reads obtained from RNAseq were aligned to the human reference genome (Homo Sapiens GRCh38.p12) and transcript abundances were extracted using the suite of tools: TopHat^27^, Bowtie^28^ and Cufflinks^29^ implemented in HISAT2 version 2.04. Finally, the gene abundance estimates were normalized using the Transcripts per Million reads (TPM) transformation^30^.

## Conclusions

Using data from a 8 day viral challenge study, this paper established a strong association between pre-exposure variability of cognitive function and severity of infection, as measured by total viral shedding and symptom severity after a person’s exposure to the common cold. A person’s cognitive variability over time was measured using thrice daily cognitive testing. Our results suggest that regularly collected cognitive performance markers, in combination with measures of stress and fatigue, may be useful for predicting susceptibility to severe symptom and viral shedding, with potential clinical and epidemiological application.

It is to be emphasized that the proposed cognitive performance variability (CPV) score is a fixed function without any tunable parameters. Such a parameter-free score does not require fitting a model to the population, unlike regression-based scores. However, if we had access to a larger sample population or a longer baseline for training, it is possible that we could improve on the CPV score by introducing some parameters. For example, we might fit a regression model with variable selection to the population, selecting the most important NCPT variables along with the regression coefficients. As another example, with a longer baseline, a temporal dependency weighted CPV model might be fitted to each participant, e.g., accounting for the effects of learning curves and circadian fluctuations.

## Supplementary Information

Supplementary Information.

## Data Availability

The data presented in this paper have been made publicly available at the University of Michigan—Deep Blue Data repository^17^ (https://doi.org/10.7302/90mc-9h22). In addition to the processed RNAseq data included in the repository, the paired-end RNAseq FASTQ files are available on the Gene Expression Omnibus^31^ (GEO accession # GSE215087 www.ncbi.nlm.nih.gov/geo/query/acc.cgi?acc=GSE215087).

## References

[1] Salthouse TA (2007). Implications of within-person variability in cognitive and neuropsychological functioning for the interpretation of change. Neurpsychology.

[2] Shipley BA, Der G, Taylor MD, Deary IJ (2006). Cognition and all-cause mortality across the entire adult age range: Health and lifestyle survey. Psychosom. Med..

[3] Haynes BI, Bauermeister S, Bunce D (2017). A systematic review of longitudinal associations between reaction time intraindividual variability and age-related cognitive decline or impairment, dementia, and mortality. J. Int. Neuropsychol. Soc..

[4] Kipnis J, Gadani S, Derecki NC (2012). Pro-cognitive properties of t cells. Nat. Rev. Immunol..

[5] Serre-Miranda C (2020). Cognition is associated with peripheral immune molecules in healthy older adults: A cross-sectional study. Front. Immunol..

[6] Taquet M, Luciano S, Geddes JR, Harrison PJ (2021). Bidirectional associations between covid-19 and psychiatric disorder: Retrospective cohort studies of 62 354 covid-19 cases in the usa. Lancet Psychiatry.

[7] Morrison GE, Simone CM, Ng NF, Hardy JL (2015). Reliability and validity of the neurocognitive performance test, a web-based neuropsychological assessment. Front. Psychol..

[8] Ando K (2010). Human lactoferrin activates nf-$$\kappa$$b through the toll-like receptor 4 pathway while it interferes with the lipopolysaccharide-stimulated tlr4 signaling. FEBS J..

[9] Stelzer G (2016). The genecards suite: From gene data mining to disease genome sequence analyses. Curr. Protoc. Bioinform..

[10] Lee J-W (2016). Orphan gpr110 (adgrf1) targeted by n-docosahexaenoylethanolamine in development of neurons and cognitive function. Nat. Commun..

[11] Cesarini L (2009). Cognitive profile of disorders associated with dysregulation of the ras/mapk signaling cascade. Am. J. Med. Genet. A.

[12] Jacobsen H, Klein SL (2021). Sex differences in immunity to viral infections. Front. Immunol..

[13] Giurgea LT (2022). Sex differences in influenza: The challenge study experience. J. Infect. Dis..

[14] Salthouse TA, Nesselroade JR, Berish DE (2006). Short-term variability in cognitive performance and the calibration of longitudinal change. J. Gerontol. B Psychol. Sci. Soc. Sci..

[15] Woods, C. *IRB pro00061238 Application: Baseline Bio-Molecular Models to Predict Infectious Disease Susceptibility, Approved by Duke Health Institutional Review Board* (2015).

[16] Turner, R. *IRB hsr 17964 Application: Baseline Bio-Molecular Models to Predict Infectious Disease Susceptibility, Approved by University of Virginia Health Sciences Research Institutional Review Board (HSR-IRB)* (2015).

[17] Hero, A. O. *et al.**Human Challenge Study Dataset 2015. University of Michigan—Deep Blue Data, Deposit ID w6634400v*. https://deepblue.lib.umich.edu/data/concern/data_sets/w6634400v (2022).

[18] Turner RB (1999). Efficacy of tremacamra, a soluble intercellular adhesion molecule 1, for experimental rhinovirus infection: A randomized clinical trial. J. Am. Med. Assoc..

[19] Zaas AK (2009). Gene expression signatures diagnose influenza and other symptomatic respiratory viral infections in humans. Cell Host Microbe.

[20] Huang Y (2011). Temporal dynamics of host molecular responses differentiate symptomatic and asymptomatic influenza a infection. PLoS Genet..

[21] Smith AP (2012). Effects of the common cold on mood, psychomotor performance, the encoding of new information, speed of working memory and semantic processing. Brain Behav. Immun..

[22] Krupp LB, LaRocca NG, Muir-Nash J, Steinberg AD (1989). The fatigue severity scale: Application to patients with multiple sclerosis and systemic lupus erythematosus. Arch. Neurol..

[23] Cohen S, Kamarck T, Mermelstein R (1983). A global measure of perceived stress. J. Health Soc. Behav..

[24] Smith CS, Reilly C, Midkiff K (1989). Evaluation of three circadian rhythm questionnaires with suggestions for an improved measure of morningness. J. Appl. Psychol..

[25] Randler C (2009). Validation of the full and reduced composite scale of morningness. Biol. Rhythm. Res..

[26] Scheffe H (1999). The Analysis of Variance.

[27] Trapnell C, Pachter L, Salzberg SL (2009). Tophat: Discovering splice junctions with rna-seq. Bioinformatics.

[28] Langmead B, Trapnell C, Pop M, Salzberg SL (2009). Ultrafast and memory-efficient alignment of short dna sequences to the human genome. Genome Biol..

[29] Trapnell C (2010). Transcript assembly and quantification by rna-seq reveals unannotated transcripts and isoform switching during cell differentiation. Nat. Biotechnol..

[30] Wagner GP, Kin K, Lynch VJ (2012). Measurement of mrna abundance using rna-seq data: Rpkm measure is inconsistent among samples. Theory Biosci..

[31] Zhai, Y. *et al.**Biochronicity Challenge Study Gene Expression Data. Gene Expression Omnibus (GEO) Accession Number GSE215087*. www.ncbi.nlm.nih.gov/geo/query/acc.cgi?acc=GSE215087 (2022).

