# Supplementary material for: Pre-exposure cognitive performance variability is associated with severity of respiratory infection
Source: Sci Rep. 2022 Dec 30;12:22589. doi: 10.1038/s41598-022-26081-6 (PMC9801154; doi:10.1038/s41598-022-26081-6)
Supplement: Supplementary file 1 — Supplementary Information. [file 41598_2022_26081_MOESM1_ESM.pdf]

Pre-exposure cognitive performance variability is associated with severity of respiratory infection: Supplementary materials

Yaya Zhai[1], P. Murali Doraiswamy[2], Christopher W. Woods[3], Ronald B. Turner[4], Thomas W. Burke[3], Geoffrey S. Ginsburg[5], and Alfred O. Hero[6,*]

[1]Dept. of Computational Medicine and Bioinformatics, University of Michigan, Ann Arbor MI 48109
[2]Departments of Psychiatry and Medicine, Duke University School of Medicine, Durham, North Carolina 27705
[3]Duke Center for Applied Genomics and Precision Medicine, Duke University Medical Center, Durham, North Carolina 27708
[4]Dept. Pediatrics, University of Virginia School of Medicine, Charlottesville, Virginia 22908
[5]All of Us Research Program, National Institutes of Health, Bethesda, MD 20892
[6]Dept. of Electrical Engineering and Computer Science, Dept. of Biomedical Engineering, and Dept. of Statistics, University of Michigan, Ann Arbor Michigan 48109
[*]hero@umich.edu

Supplementary Materials

Definitions of NCPT features used in analysis

The cognitive tests were performed in a series of 18 to 25 sessions over the duration of of the challenge study (Fig. S12). Except for a single measure that we selected as a control, `trail-layouNum`, we eliminated all of the measures that were deemed unaffected by cognitive performance, e.g., software generated quantities numbering the sessions and others used to randomly initialize test layouts. The remaining measures, with some modifications explained below, were adopted as the 18 NCPT variables used in our analysis.

The four cognitive tests taken by the participants during the sessions are clinical tests called “Digital symbol coding," “Go No Go," “Trail making B," and “Posner cuing." The 18 cognitive measures used in the analysis are defined below

- Digit symbol coding test. This test assesses processing speed, working memory, visuo-spatial processing, and attention.
  1. `digSym-correct`: Total number of correct trials in 90 seconds; this is the primary outcome for this assessment
  2. `digSym-error`: Total number of incorrect trials in 90 seconds
  3. `digSym-time`: Average reaction time in milliseconds across all trials (so including both incorrect and correct trials)
- Go No Go. This test assesses response inhibition and processing speed. Participants are required to respond as quickly as possible to a target stimulus while avoiding responding to distractors.
  4. `reaction-error`: Total number of incorrect trials out of 10 trials; 3 errors in a row ends the assessment.
  5. `reaction-time`:Average reaction time in milliseconds for Go trials; this is the primary outcome for this assessment.
- Trail Making B. This test assesses visual ability, motor functioning, and cognitive processes in a timed trial. Often used to screen for dementia by assessing cognition and memory.
  6. `trail-layoutNum`: Numeric value corresponding to the specific layout of items on the screen; chosen randomly at the beginning of the assessment and not dependent on the participant’s responses (a neutral control variable).
  7. `trail-tutorialTime`: Time in milliseconds to complete the tutorial.
  8. `trail-error`: Total number of errors during the assessment.
  9. `trail-time`: Total time in milliseconds to complete the assessment; this is the primary outcome for this assessment.

- Posner Cueing. This test assesses a person's ability to perform spatial attentional shift. Often used to assess focal brain injury and its affect on spatial attention.

10. `posner-tutorialError`: Number of errors made during the tutorial.
11. `posner-tutorialTime`: Time in milliseconds to complete the tutorial.
12. `posner-correct`: Number of correct trials out of a total of 100.
13. `posner-responseTime`: Average reaction time in milliseconds across all 100 trials 14 posner-timeCongruent Average response time in milliseconds across all congruent trials; a congruent trial is defined as the cue and the stimulus pointing in the same direction.
15. `posner-timeIncongruent`: Average response time in milliseconds across all incongruent trials; an incongruent trial is defined as the cue and the stimulus are pointing in opposite directions.
16. `posner-totalTime`: Total time in milliseconds to complete full assessment (100 trials).
17. `posner-Ncongruent`: The number of congruent trials shown during assessment. Derived from Lumos NCPT variables provided.
18. `posner-correctCongruent`: The number of correct responses to congruent trials. Derived from Lumos NCPT variables provided.

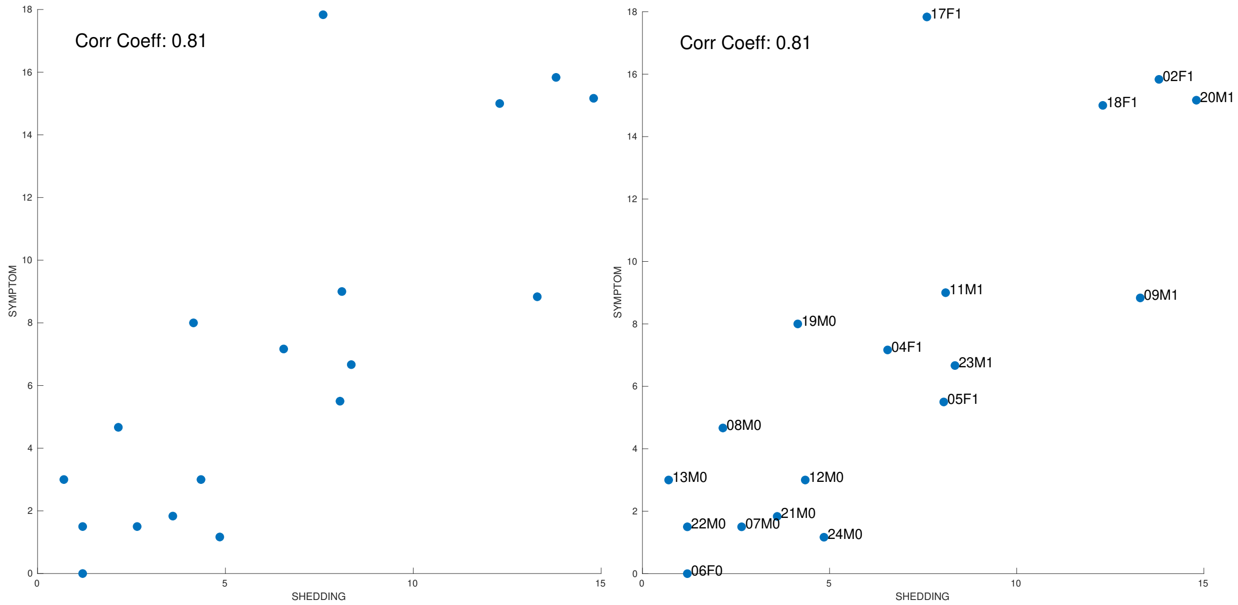

Figure S1. Total symptom severity is correlated with total shedding titers (0.81 Pearson correlation with pvalue 5×10^{-5}). Left: scatterplot of shedding vs symptom for the 18 subjects in the challenge study. Right: same scatterplot of with subject ID annotations. With one exception (subject ID 19M0), the low shedding subjects complain of less severe symptoms than do the high shedding subjects. The level of shedding of subject 19M0 is near the middle of the low range (Fig. 3) and his symptom score is near the mean (7.0) of the overall symptom distribution. As shedding was measured once daily while symptoms were reported multiple times per day, shedding is computed as the sum of the all post-incoculation shedding titrations while symptom is computed as the sum of the daily averaged modified Jackson scores over the post-inoculation time period of the study.

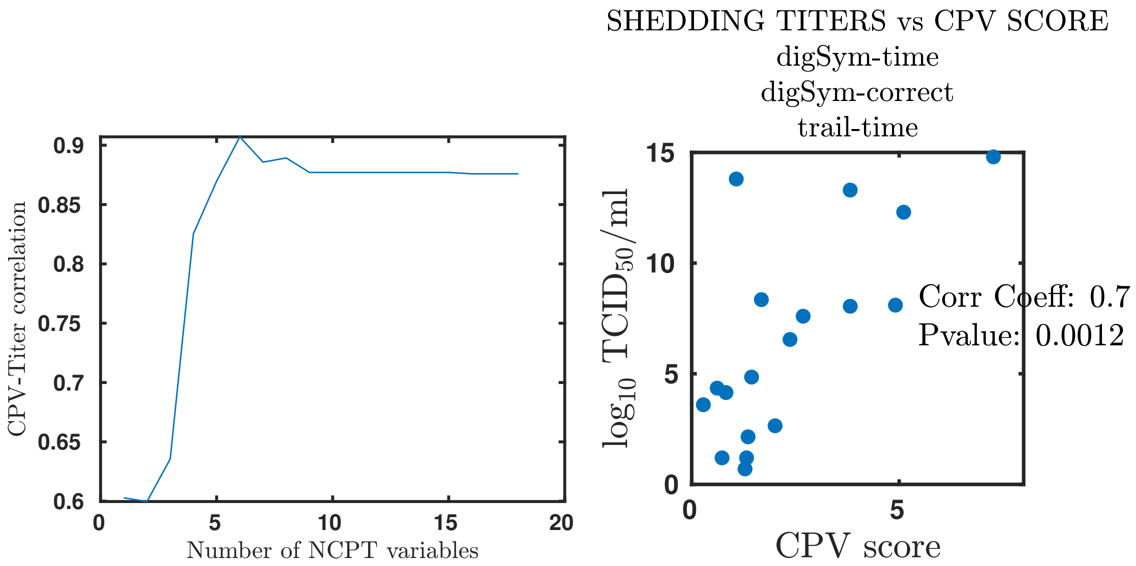

Figure S2. Left: plot of CVP-titer correlation obtained after progressive pruning one-by-one the NCPT measures, ordered as in Fig. 1c, composing the CVP score (maximum of the univariate CVP's defined for each NCPT measure). The combination of 6 NCPT measures digSym-time, `digSym-correct`, `reaction-time`, `posner-tutorialTime`, `trail-time` and `trail-tutorialTime` in the CPV score achieves a correlation of greater than 0.9. Right: scatterplot of CVP score with only the three variables digSym-time, `digSym-correct`, and `trail-time`. The CVP score composed of only these three NCPT measures is correlated to titers at a level greater than 0.7. Addition of a fourth NCPT variable `reaction-time` into the CPV score achieves approximately the same level of correlation (0.69, data not shown here).

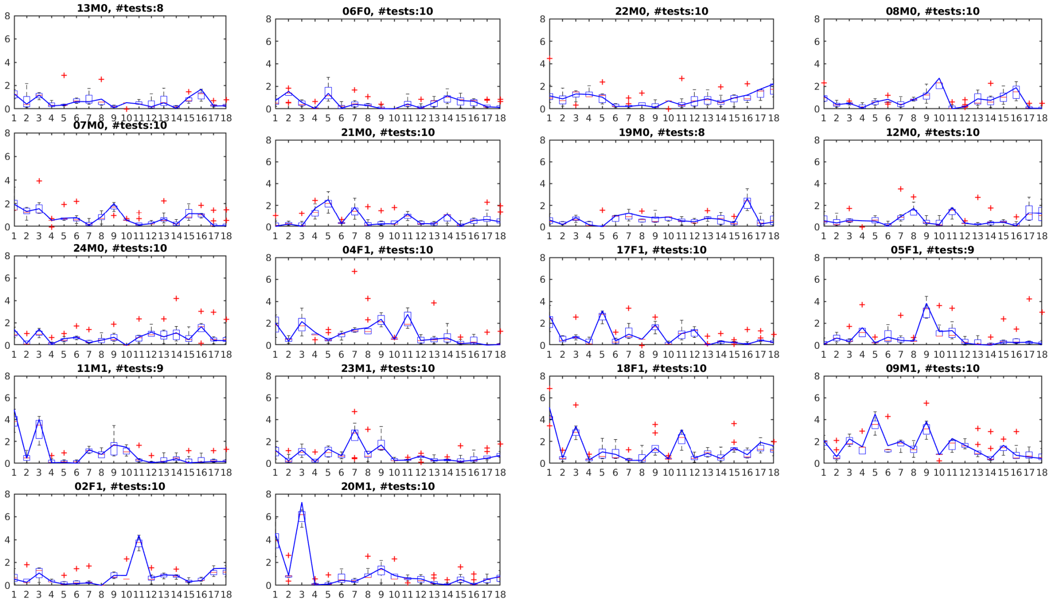

Figure S3. Boxplots of the univariate Cognitive Performance Variability scores over the 18 NCPT variables in Figure 1b for all participants. Plots are arranged from lowest shedding (top left) to highest shedding (bottom right). challenge study participants. The Solid blue curve denotes the CPV scores computed for each NCPT variable using the full session sequence, but omitting the initial screening session. Red boxplots show the distribution of the leave-one-out (loo) scores computed by successively dropping a single session from the sequence of post-screening sessions.

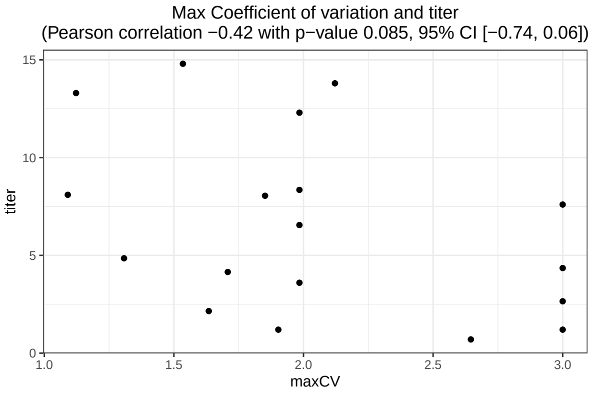

(a) Coefficient of variation and titer.

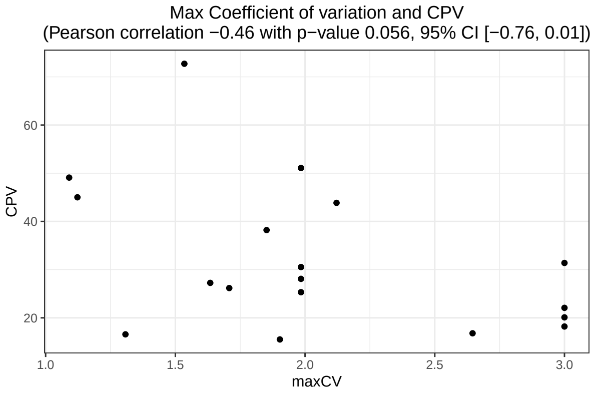

(b) Coefficient of variation and CPV.

Figure S4. Coefficient of variation was computed for baseline NCPT variables as the ratio of the standard deviation to the mean[1]. (a) Coefficient of variation does not have a significant correlation with viral shedding, and the correlation coefficient is about the half compared to the correlation between CPV and viral shedding. (b) The correlation between coefficient of variation and CPV is about -0.46.

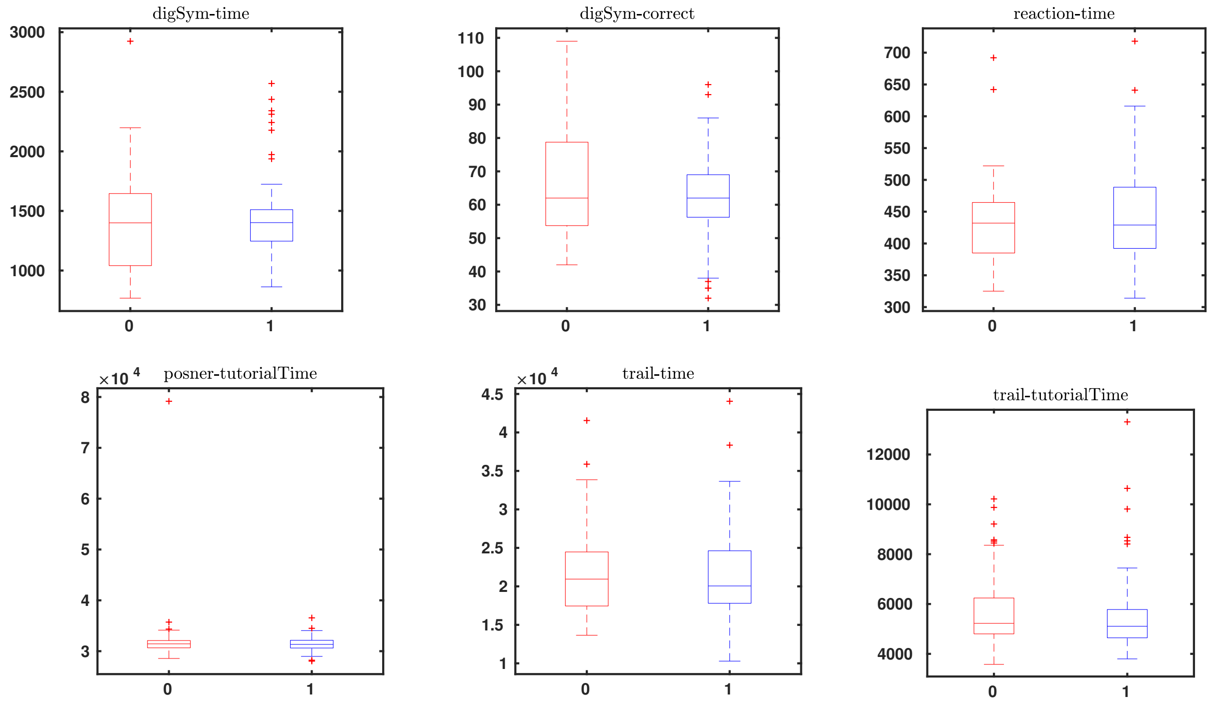

Figure S5. Boxplots of the 6 NCPT measures digSym-time, `digSym-correct`, `reaction-time`, `posner-tutorialTime`, `trail-time` and `trail-tutorialTime` for for low shedding (class 0) and high (class 1) study participants over baseline. There is no discernable class difference between the marginal distributions for any of these measures. This is to be contrasted to the proposed CPV score, which is a longitudinal statistic for detecting a trend over time (Fig. S6).

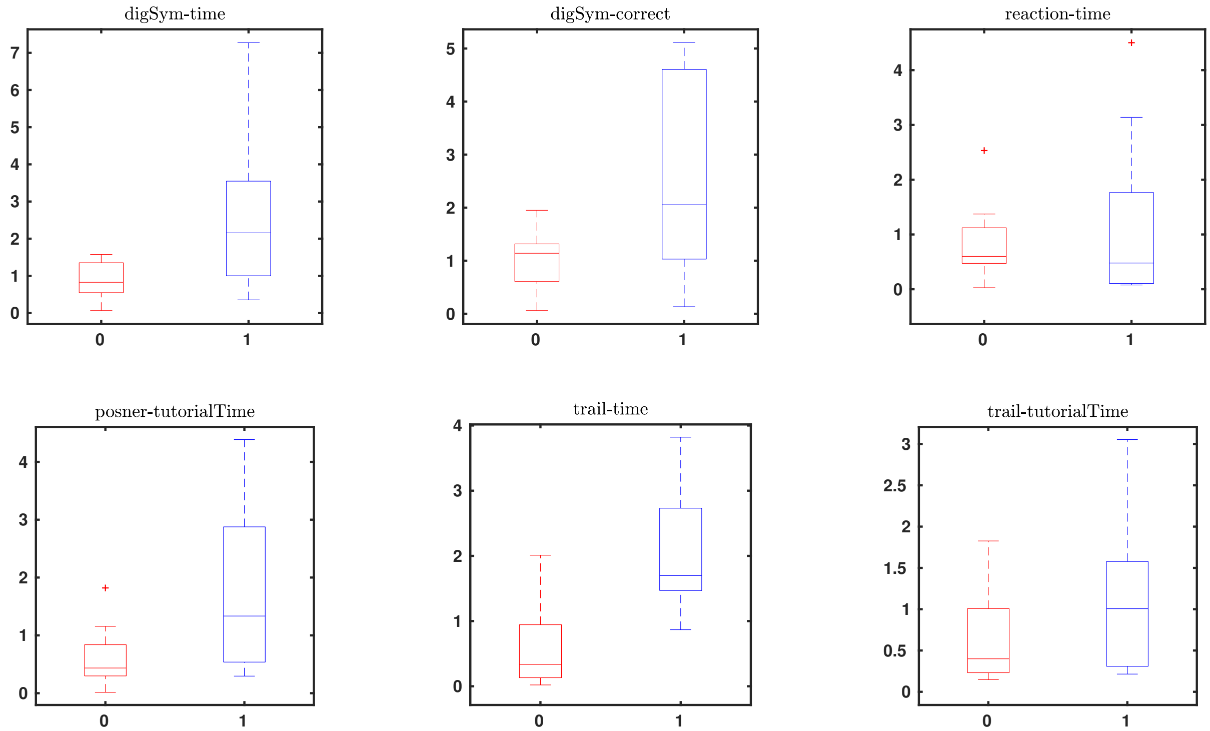

Figure S6. Boxplots of the univariate CPV for each of the 6 NCPT measures digSym-time, `digSym-correct`, `reaction-time`, `posner-tutorialTime`, `trail-time` and `trail-tutorialTime` for for low shedding (class 0) and high (class 1) study participants over baseline. While no single univariate CPV measure is sufficient to separate the classes, the CPV score (max of univariate CPVs) can.

Variable 1	Variable 2	ρ	p_value	95% CI
CPV score	titer	0.88	1.90E-06	[0.69,0.95]
CPV score	PSS	0.62	5.73E-03	[0.22,0.82]
CPV score	duration_sd	-0.5	3.91E-02	[-0.79,-0.03]
CPV score	vafs_sd	0.48	4.27E-02	[0.02,0.77]
CPV score	vafs_mean	-0.46	5.3E-02	[-0.76,0.01]
vafs_mean	titer	-0.51	2.98E-02	[-0.79,-0.06]

Table S1. Table of statistically significant correlations between cognitive biomarkers and shedding titers over baseline (Fisher test at level 0.05). Except for shedding (titer), all quantities listed in the table were computed based on baseline samples only.

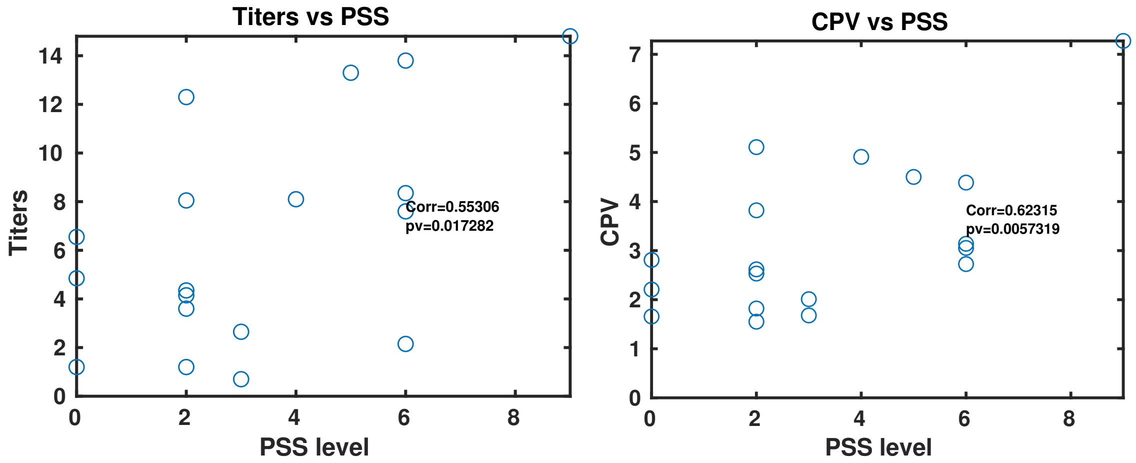

Figure S7. Scatter plots showing association between cognitive data collected at screening, the perceived stress scale (PSS) with the amount of viral shedding (left) and with the pre-exposure cognitive performance variability (CPV) over baseline.

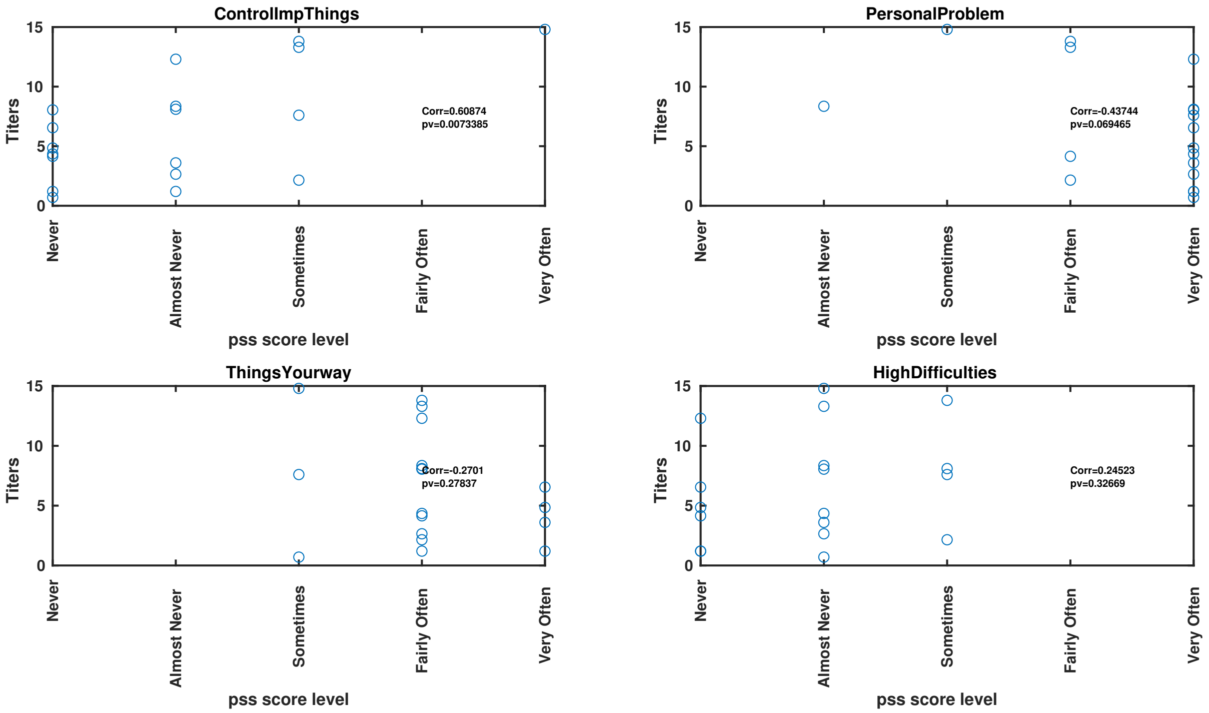

Figure S8. Scatter plots of viral shedding and four perceived stress variables self-reported by study participants during screening.

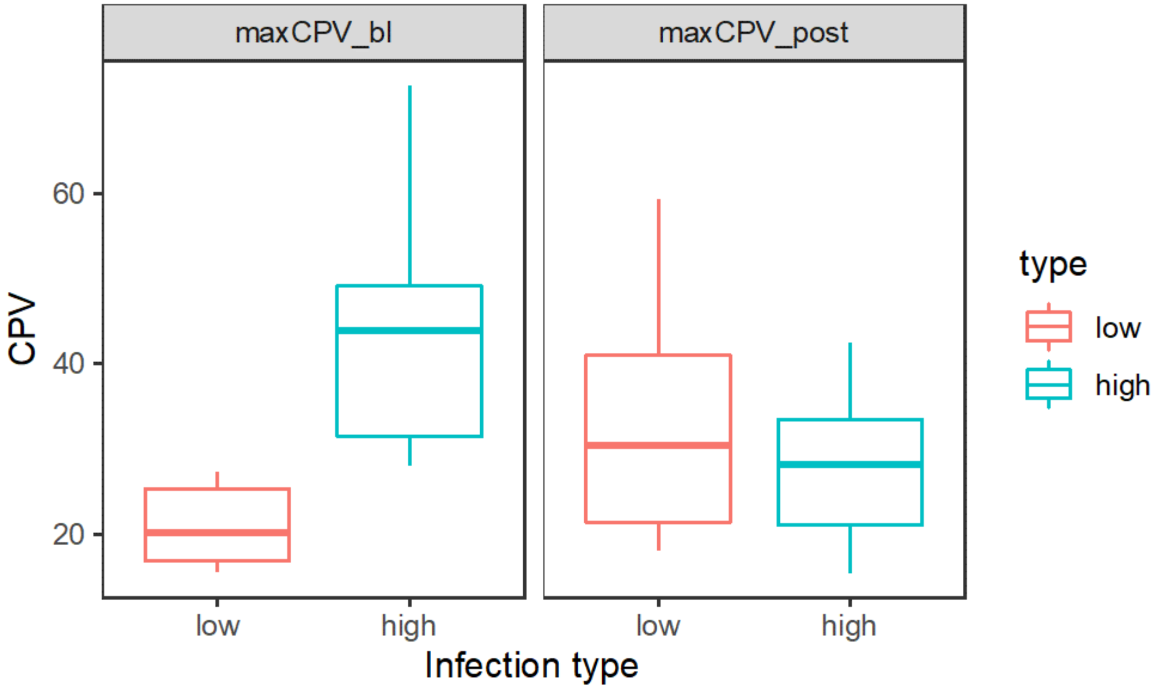

Figure S9. Boxplots of CPV for low vs high shedders over baseline (left) and post-inoculation (right). The significant difference between CPV of low and high shedders at baseline disappears after inoculation.

		Low shedder	High shedder	Total
Median Age (range)		21 (20-24)	20 (19-33)	20(19-33)
Gender	Male	8	4	12
	Female	1	5	6
Ethnicity	White	7	7	14
	Black	2	0	2
	Asian	0	2	2
Dominant hand	Right	9	9	18
	Left	0	0	0
Median Systolic BP (range)		120 (100-130)	110(100-120)	112(100-130)
Median Diastolic BP (range)		70 (60-80)	70 (58-79)	70 (58-80)
Median Pulse (range)		64 (60-72)	72 (60-80)	67 (60-80)
Median Respiratory Rate (range)		20 (16-20)	18 (16-20)	19 (16-20)
Median Weight (range)		71 (61-89)	65 (55-73)	67 (55-89)
Getup time	before 6:30	0	1	1
	6:30-7:45	1	0	1
	7:45-9:45	5	6	11
	9:45-11	3	1	4
	11-12	0	1	1
Bed time	10:15-12:30	5	5	10
	12:30-1:45	4	2	6
	1:45-3	0	2	2
Productive time	Morning	4	3	7
	Afternoon	3	1	4
	Evening	2	3	5
	Night	0	2	2

Figure S10. Study demographics (upper 3 rows), physiological statistics, and self-reported biochronicity (morningness) statistics (lower 3 rows) collected during screening for the 18 subjects included in our analysis.

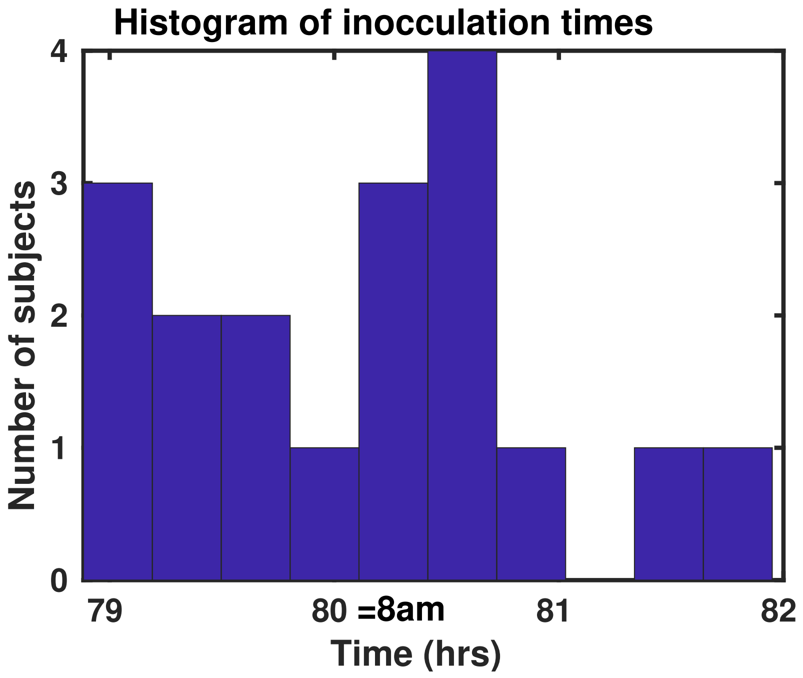

Figure S11. Histogram of the inoculation times for the 18 participants. Eighty hours corresponds to 8am on the morning of the 4th day of the challenge study. All but 2 participants are inoculated between 7 and 9am.

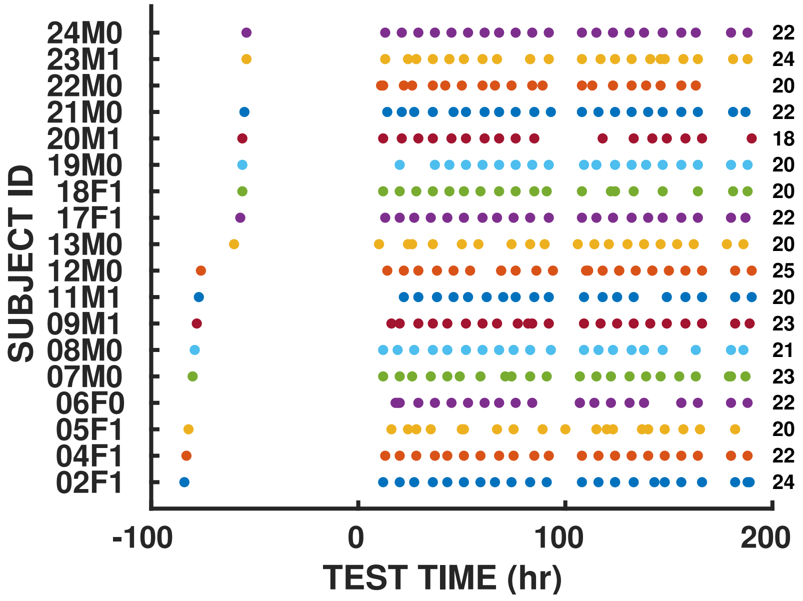

Figure S12. Timing of sessions over the entire study (pre- and post-exposure) for the 18 participants. The time axis is indexed over hours where 0h corresponds to 12AM of the first day of the challenge study and 80h corresponds approximately to the time of viral inoculation on the morning of the 4th day (8am). Figure 3b only shows the pre-exposure sessions.

References

1. Brown, C. E. Coefficient of variation. In *Applied multivariate statistics in geohydrology and related sciences*, 155–157 (Springer, 1998).

2. Ando, K. *et al.* Human lactoferrin activates nf-κb through the toll-like receptor 4 pathway while it interferes with the lipopolysaccharide-stimulated tlr4 signaling. *The FEBS journal* **277**, 2051–2066 (2010).

3. Stelzer, G. *et al.* The genecards suite: from gene data mining to disease genome sequence analyses. *Curr. protocols bioinformatics* **54**, 1–30 (2016).
